# The Pituitary-Adrenal Response to Paradoxical Sleep Deprivation Is Similar to a Psychological Stressor, Whereas the Hypothalamic Response Is Unique

**DOI:** 10.3389/fendo.2022.885909

**Published:** 2022-07-08

**Authors:** Danilo A. Moraes, Ricardo B. Machado, Michael Koban, Gloria E. Hoffman, Deborah Suchecki

**Affiliations:** ^1^ Department of Psychobiology, Universidade Federal de São Paulo, São Paulo, Brazil; ^2^ Grupo de Pesquisa em Psicossomática, Universidade Ibirapuera, São Paulo, Brazil; ^3^ Department of Biology, Morgan State University, Baltimore, MD, United States

**Keywords:** acute stress, repeated stress, HPA axis, vasopressin, ACTH, corticosterone

## Abstract

Stressors of different natures induce activation of the hypothalamic-pituitary-adrenal (HPA) axis at different magnitudes. Moreover, the HPA axis response to repeated exposure is usually distinct from that elicited by a single session. Paradoxical sleep deprivation (PSD) augments ACTH and corticosterone (CORT) levels, but the nature of this stimulus is not yet defined. The purpose of the present study was to qualitatively compare the stress response of animals submitted to PSD to that of rats exposed once or four times to cold, as a physiological stress, movement restraint (RST) as a mixed stressor and predator odour (PRED) as the psychological stressor, whilst animals were submitted for 1 or 4 days to PSD and respective control groups. None of the stressors altered corticotropin releasing factor immunoreactivity in the paraventricular nucleus of the hypothalamus (PVN), median eminence (ME) or central amygdala, compared to control groups, whereas vasopressin immunoreactivity in PSD animals was decreased in the PVN and increased in the ME, indicating augmented activity of this system. ACTH levels were higher after repeated stress or prolonged PSD than after single- or 1 day-exposure and control groups, whereas the CORT response was habituated by repeated stress, but not by 4-days PSD. This dissociation resulted in changes in the CORT : ACTH ratio, with repeated cold and RST decreasing the ratio compared to single exposure, but no change was seen in PRED and PSD groups. Comparing the magnitude and pattern of pituitary-adrenal response to the different stressors, PSD-induced responses were closer to that shown by PRED-exposed rats. In contrast, the hypothalamic response of PSD-exposed rats was unique, inasmuch as this was the only stressor which increased the activity of the vasopressin system. In conclusion, we propose that the pituitary-adrenal response to PSD is similar to that induced by a psychological stressor.

## Introduction

Stress can be defined as an uncontrollable and unpredictable situation that surpasses the individual’s regulatory capacity (1). The hypothalamic-pituitary-adrenal (HPA) axis is the main neuroendocrine stress response system. Depending on the nature of the stimuli, different pathways arrive at the parvocellular region of the paraventricular nucleus of the hypothalamus (PVN), where corticotropin releasing hormone (CRH) and arginine-vasopressin (AVP) producing cells are located (2, 3). In the presence of a stressor, CRH and AVP are secreted from the median eminence, reach the anterior pituitary and stimulate the synthesis and release of adrenocorticotropic hormone (ACTH), which, in turn, activates the adrenal cortex culminating in the release of glucocorticoids (4, 5). These hormones, in turn, lead to cardiovascular and metabolic changes to prepare the individual to cope with the stressful situation (6).

Stressful stimuli are classified according to their nature, leading to different magnitudes of HPA axis activation, recruitment of brain areas and neurotransmitter systems. Therefore, the magnitude and time course of the HPA axis stress response may be used to determine the nature of the stressor (Koolhaas et al., 2011). Stressors can be categorized into physical, e.g., producing direct physiological changes to allow recovery of homeostasis; psychological or stimuli which are perceived as a threat, factual or eminent, by the individual; and mixed stressors that present both physical and psychological components (7–9). Moreover, whether stressors are applied acutely, chronically (intermittently or continuously) lead to distinct response outcomes (10). For instance, compared to other acute stressors, exposure to cold (4°C, for 1 h), a typical physical stressor, induces the greatest corticosterone (CORT) release (11), but when exposure to this stressor is repeated for four days, twice per day, the CORT levels are no longer different from control values, suggesting that habituation had taken place (12). Conversely, stimuli with a psychological component tend to produce attenuated CORT (1, 13) and higher ACTH secretion (11, 14). Importantly, few studies simultaneously compared the stress responses to different stimuli under similar experimental conditions (10–12). It is costumery to find review papers that compile different studies to make this comparison (1, 8, 15). After comparing different stress models, such as immobilization, restraint, electric foot shock and social isolation, Bali and Jaggi (15) concluded that the inconsistency in the biochemical, behavioural and physiological results are due to differences in the stress protocols, suggesting the need for standardization of the models.

Paradoxical sleep (PS; also referred to as rapid eye movement – REM sleep) deprivation (PSD), has been regarded as stressful, but its nature has not yet been characterized. Several studies show that exposure of animals to PSD results in activation of the HPA axis, regardless of the method used (16–24). The single platform method (25) is one of these methods and the data show activation of the HPA axis at its different levels. PSD increases the levels of CRH in the limbic system and pituitary and reduces hypothalamic content of CRH, reduces the binding of CRH receptor-type 1 in the pituitary (26), and increases CRH immunoreactivity in the PVN after 4 (27) or 5 (28) days. Animals subjected to 24 h of PSD have increased levels of CORT (21, 29–31), whereas 96 h of unremitting sleep deprivation increases ACTH levels, in addition to CORT (21, 24, 27, 32–35). Despite this evidence, there is still a debate on whether these changes are due to the stress associated with the method or to the loss of sleep. The inclusion of an environmental control group to overcome this problem, by means of large platforms onto which rats could sleep, has yielded controversial results, mainly because under this condition animals are sleep deprived to a lesser extent (36, 37). Thus, the aim of this study was to evaluate the pattern of HPA axis activity in animals subjected to 24 h or 96 h of PSD and indirectly compare the findings with the stress response of animals subjected to stressors of different natures (cold as physical; predator odour as psychological; and restraint as mixed stressors), applied once or four times, to determine the stress nature of PSD. Cold and restraint were chosen as physical and mixed stressors, respectively, as they are stimuli present in the single platform method to induce PSD, whereas predator odour was used as the psychological stressor not present in PSD.

Based on the knowledge that prolonged sleep deprivation in humans increases irritability and impairs emotional regulation (38), and that poor quality sleep and insomnia are triggers for depressive episodes (39), we hypothesized that the stressful nature of PSD would resemble that of mixed or psychological stress, i.e., the HPA axis’ activity induced by PSD would be closer to that of restraint stress or exposure to predator scent than it would be to temperature changes.

## Methods

### Subjects

We used male *Wistar* rats (235-450 g), 90 day-old (n = 182), from Centro de Desenvolvimento de Modelos Experimentais para Medicina e Biologia (CEDEME) of Universidade Federal de São Paulo (UNIFESP). The animals were kept in a controlled environment, with 12 h light/12 h dark cycle (lights on at 7:00 a.m.) and temperature of 23 ± 1°C. All animals were housed individually in plastic cages for three days before the onset of the experiments, which commenced only after the approval of the University’s ethical committee (CEUA No. 1871160914).

### Experiments/Groups

*Experiment 1:* The animals were distributed in 8 groups, based on the stress procedure and the frequency to which they were exposed, single or repeated: home cage (HC); cold stress (COLD); restraint (RST); predator odour (PRED).

*Experiment 2:* The animals were distributed in 6 groups, based on the housing and length of the procedure, 1 day or 4 days: container control (CC, used as an equivalent to the home-cage control); large platform (L-PLAT); small platform (S-PLAT).

In both experiments, each group consisted of 13 animals, of which eight were decapitated and 5 were perfused. The repeated or 4 day-long protocols started three days before the single stress protocol, so that all animals were euthanized on the same day.

### Stress Procedures

#### Cold

Animals were placed individually in metal wire cages inside a freezer set at -4°C ± 2°C with the door partially opened for ventilation, for 1 h. The temperature was chosen based on the activation of areas related to physical stress (8). Exposure to cold began at 08:00 a.m.

#### Restraint

The animals were kept in plastic cylinders (21 cm length x 6 cm diameter) for 1 h (Palma et al., 2000), beginning at 08:00 a.m. One end of the cylinder was closed with wire mesh and after the animal entered the cylinder a wooden door (with a small hole to accommodate the tail) closed the other end. Importantly, rats of similar size were used to make sure that they were similarly restricted.

#### Predator Scent

Each animal was taken to a separate room from the animal facility and exposed, in its own cage, to a petri dish containing approximately 60 g of sand with cat urine. This sand was obtained from the litter box of two male domestic cats used during the two days prior to the experiment and the feces were removed from the sand. Exposure to predator odour began at 08:30 h and lasted for 30 min. In the protocol of repeated exposure, the animals were housed in a clean cage, after the end of each session.

#### Paradoxical Sleep Deprivation

PSD was performed by the single platform method. Each rat was housed in one container (22 cm length x 22 cm width x 35 cm height) filled with water, onto a platform with 6.5 cm (small platform) in diameter (Cohen & Dement, 1965). The platform remained immersed in water up to 1.0 cm from its top edge. Food (side feeder) and water (located in the upper part of the container) were available *ad libitum*. The PSD control group was accommodated in the same container as PSD rats, but on platforms with 14.0 cm (large platform) in diameter, to prevent them from falling into the water during PS, although previous data show that there is also considerable loss of sleep and a subsequent PS rebound in the recovery period (Machado et al., 2004). The control group was kept in the same experimental environment as the other groups, but instead of water, the container was lined with corn cob shavings. The animals were habituated to their experimental environments for 1 h/day for three days before the beginning of the experiments. PSD started at 09:00 a.m. and lasted 24 h or 96 h.

### Body Weight

Animals were weighed before the beginning (initial weight) and after the last stress session (final weight). The percentage of body weight change was calculated by the following equation: [(final weight - initial weight)/initial weight] x 100.

### ACTH and CORT Plasma Concentrations/CORT : ACTH Ratio

Immediately after the end of the stress protocols, one subset of animals (n = 8/group) was decapitated for collection of trunk blood in tubes containing 0.1 mL of 6% ethylenediaminetetraacetic acid (EDTA). The blood was centrifuged at 1316 g for 20 min at 4°C and the plasma was separated into two aliquots. ACTH concentrations were determined by enzyme-linked immunosorbent assay (ELISA) (Phoenix Pharmaceuticals, Inc., USA). The test was performed according to the instructions provided by the manufacturer’s catalogue. The sensitivity of the test is 0.08 ng/mL, ranging from 0 to 25 ng/mL; the intra-assay variation is <10% and inter-assay variation, <15%. CORT concentrations were determined by mass spectrometry method (Waters^®^ QuatroMicro, Kinetex^®^ column 50 x 2.10 mm, 2.6 μm, mobile phase methanol/water, flow 0.2 mL/min). CORT : ACTH ratio was calculated by dividing the CORT plasma concentration by the ACTH plasma concentration, to estimate the amount of ACTH that would result in the CORT output.

### Immunocytochemistry

Immediately after the end of each stress procedure, the remaining animals (n = 5/group) were anesthetized with thiopental. After thoracic opening and clamping of the abdominal aorta, cardiac puncture was performed and the perfusion by gravity of 150 mL of saline solution with heparin (to wash the brain of the animal) started. Subsequently, 150 ml of formalin solution (4% buffered formaldehyde) was infused. After fixation, the entire head was immersed in formalin solution for 24 h at 4°C. Then, the brains were removed from the skull and immersed in 30% sucrose solution, until they sank. Next, the brains were dried to remove all excess sucrose solution, immersed in cryostat embedding solution (Tissue Tek), frozen in isopentane (-80°C) and stored in the freezer (-80°C) for later processing. All immunocytochemical reactions were performed in the Neurobiology Laboratory at Morgan State University, under supervision of Dr. Gloria Hoffman, using the Pelco BioWave Pro histological microwave with SteadyTemp recirculating water bath, following the protocol described below (40). The brains were cut into 30 μm slices in the microtome, which were kept in wells filled with antifreeze solution (sucrose, ethylene glycol and polyvinylpyrrolidone) (Watson et al., 1986) and stored at -20°C for subsequent free-floating immunocytochemical techniques. Briefly, the sections were rinsed multiple times in phosphate buffered saline (PBS) and then incubated with rabbit anti-CRH (1:100,000) (PBL # rC68, gift from Dr. Roger Guillemin, Salk Institute) or rabbit anti-AVP (1:300,000) (Miles; cat: 647171) in PBS with 0.4% Triton X-100 for 1 h at room temperature on a rotator at 55 rpm and subsequent for 48 h at 4 C. After a multiple (3–5) rinses in PSB, the sections were incubated for 19 min at room temperature in biotinylated goat anti-rabbit IgG (Abcam; cat: ab6720) at a dilution of 1:600 in PBS with 0.4% Triton X-100, rinsed again in PBS, and then incubated in avidin-biotin complex solution (VECTASTAIN Elite ABC HRP Kit; cat: PK-6100) diluted in PBS with 0.4% Triton X-100 at room temperature for 19 min. Next, the tissues were rinsed in PBS and then Tris buffer (pH 7.2). NiDAB (DAB Peroxidase HRP Substrate Kit, with Nickel, 3,3’-diaminobenzidine; cat: SK-4100) was used for revelation (20 min of staining time). The sections were rinsed in Tris buffer to stop the reaction and mounted onto gelatin-subbed slides. The slides were dried overnight, dehydrated through alcohols, cleared with xylene, and cover-slipped.

The images were captured by a 10x objective of a microscope equipped with a digital camera (Olympus DP71, Japan). Immunoreactivity (ir) was quantified bilaterally using ImageJ software (National Institute of Health) and represented by the percentage of the region of interest (% ROI): [(labelled area/total area] x 100. Brain areas were anatomically defined according to Paxinos and Watson atlas of the rat brain (41): PVN (bregma – 1.92 mm), central amygdala (CeA) (bregma – 2.04 mm) and median eminence (ME) (bregma – 2.28 mm). In the ME, CRH and AVP %ROI was determined in the external zone. Moreover, AVP, %ROI was calculated separately for the magno- and the parvocellular parts of the PVN.

### Statistical Analysis

Statistical analysis was performed with the software *Statistica 12.0* (StatSoft^®^, USA). For all analyses and separately for each experiment, two-way ANOVA was applied, with factors Group (Home Cage [HC], Cold [COLD], Restraint [RST], Predator Odour [PRED]) and Frequency (Single, Repeated) for Experiment 1, and Group (Container Control [CC], Large Platform [L-PLAT], Small Platform [S-PLAT]) and Length (1 day, 4 days) for Experiment 2. When appropriate, Newman-Keuls *post hoc* test was used, and the level of significance was set at p < 0.05. Pearson’s correlation test was used to assess the correlations between CRH-ir in the PVN and CeA.

## Results

### Body Weight

ANOVA revealed main effects of Group (F_3,96_ = 12.66, p < 0.001) and Frequency (F_1,102_ = 8.37, p < 0.005). All stressors decreased body weight gain compared to HC (p < 0.05) and COLD resulted in the largest reduction, compared to all other stressors (p’s < 0.005). Moreover, body weight gain was lower after repeated than after single exposure (p < 0.005).

**Figure 1:**
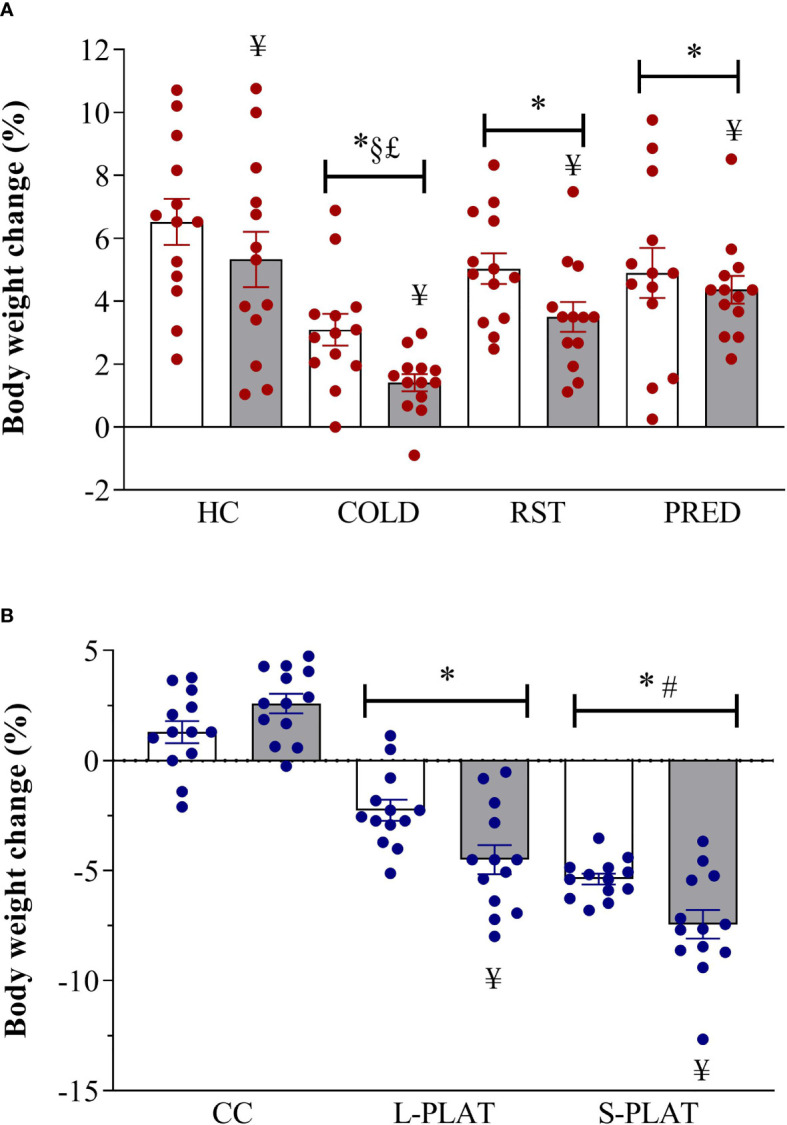
**(A)** Effect of different stressors and **(B)** of PSD on body weight change. Data were plotted with individual values and the columns represent the mean ± SEM of 13 animals/subgroup. HC, home cage; RST, restraint stress; PRED, predator odour; CC, container control; L-PLAT, large platform; S-PLAT, small platform. White columns represent single or 1-day exposure and grey columns, repeated or 4-day exposure. * = different from respective control group; § = different from RST group; £ = different from PRED group; # = different from L-PLAT group; ¥ - different from single or from 1-day exposure.

For the PSD study, there were main effects of Group (F_2,72_ = 133.34; p < 0.001), Length (*F*_1,72_ = 5.63, p < 0.05) and a Group x Length interaction (F_2,72_ = 7.39, p < 0.005). Regardless of the length of deprivation, animals from S-PLAT group lost more weight than the those of the CC and L-PLAT groups (p’s < 0.0005), whereas L-PLAT rats also showed a significant body weight loss compared to their respective CC groups (p < 0.0005). Except for CC rats, four-day exposure to the deprivation environment caused greater body weight loss than one day (p’s < 0.01). These results are shown in **Figure 1**.

### AVP-ir

**Figure 2:**
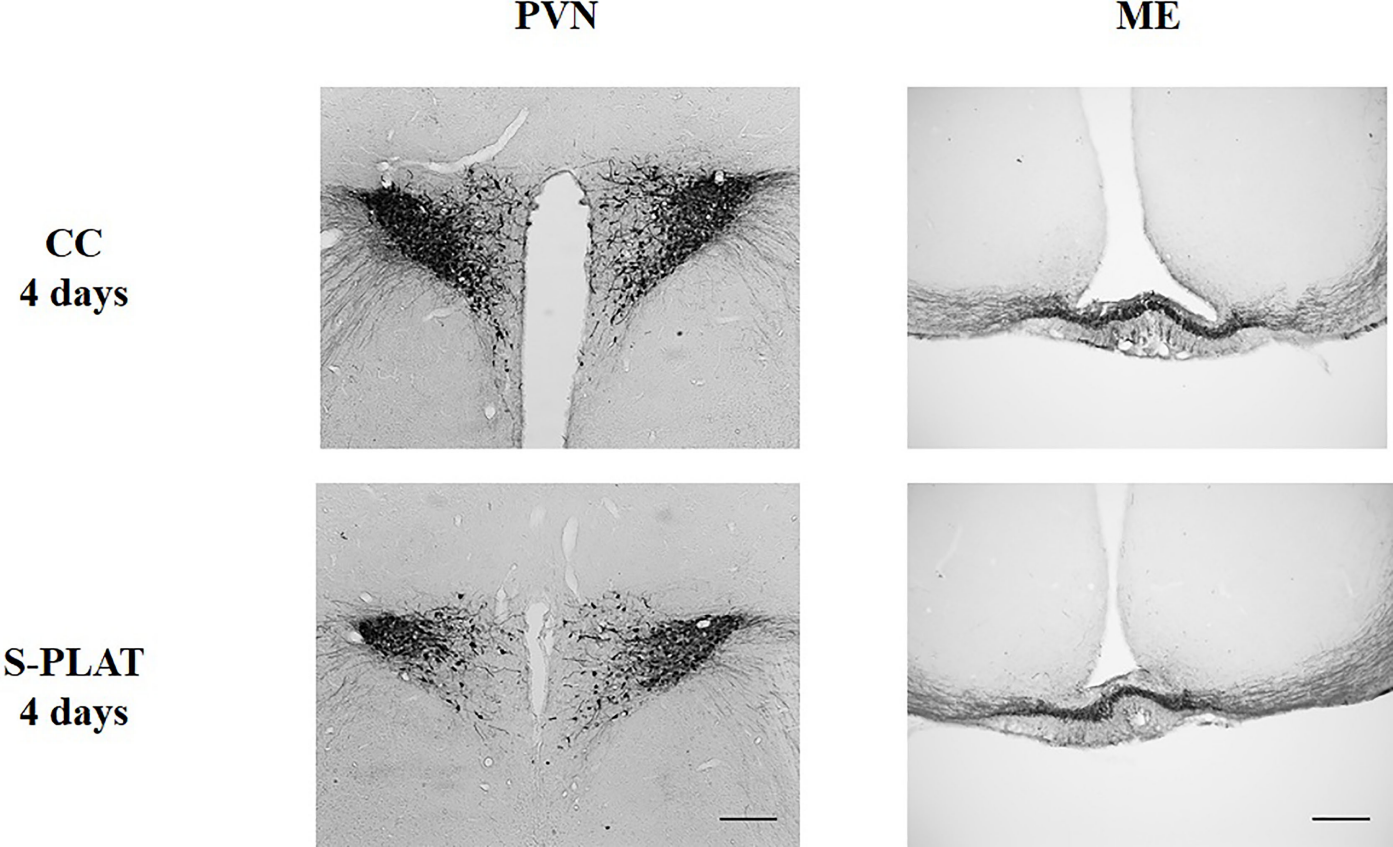
Representative photos of AVP immunocytochemistry in the PVN (left) and in the external zone of the ME (right) of one control (CC) and one rat submitted to 4 days of paradoxical sleep deprivation (S-PLAT). Scale bar = 200 μm.

There was considerable attrition of integral sections in the ME to allow a reliable assessment of %ROI, resulting in small sample size in some groups. In the PVN, there were no differences between magno- and parvocellular parts; therefore, the results are reported for the pooled quantification of AVP %ROI **Figure 2**.

**Figure 3:**
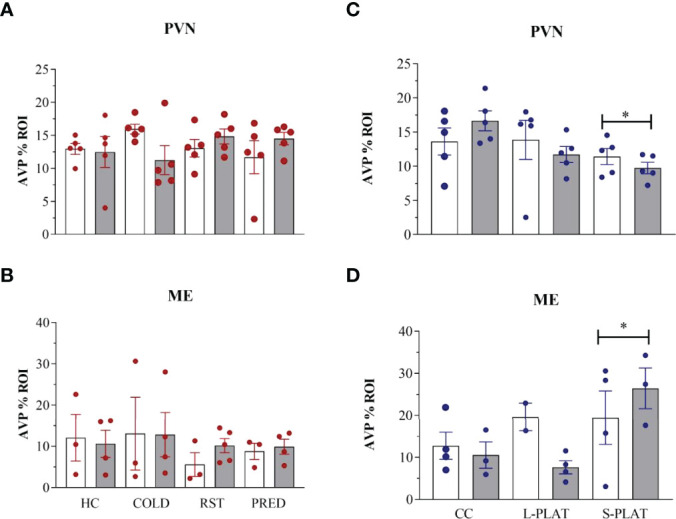
AVP-ir in the PVN and external zone of the ME after several stressors **(A, B)** or after PSD **(C, D)**. Quantification of staining is shown for each individual and the columns represent the mean ± SEM of 2 to 5 rats/subgroup. HC, home cage; RST, restraint stress; PRED, predator odour; CC, container control; L-PLAT, large platform; S-PLAT, small platform. White columns represent single or 1-day exposure and grey columns, repeated or 4-day exposure. * = different from respective control group.

There were no stressors’ effects on AVP-ir in the PVN (F_3,32_ = 0.21, p = 0.88) or in the ME (F_3,21_ = 0.57, p = 0.64) (**Figures 3A**, **3B**, respectively). Likewise, no frequency effect [PVN (F_1,32_ = 0.01, p = 0.90), ME (F_1,21_ = 0.11, p = 0.74)] or interaction between the factors [PVN (F_3,32_ = 2.05, p = 0.13), ME (F_3,21_ = 0.20, p = 0.89)] were detected.

There was a main effect of Group in the PVN (F_2,24_ = 3.52, p < 0.05) (**Figure 3C**) and the ME (F_2,14_ = 3.98, p < 0.05) (Fig. 3D), as animals from S-PLAT group showed less AVP-ir in the PVN and more AVP-ir in the ME than CC group (p < 0.05).

### CRH-ir

**Figure 4:**
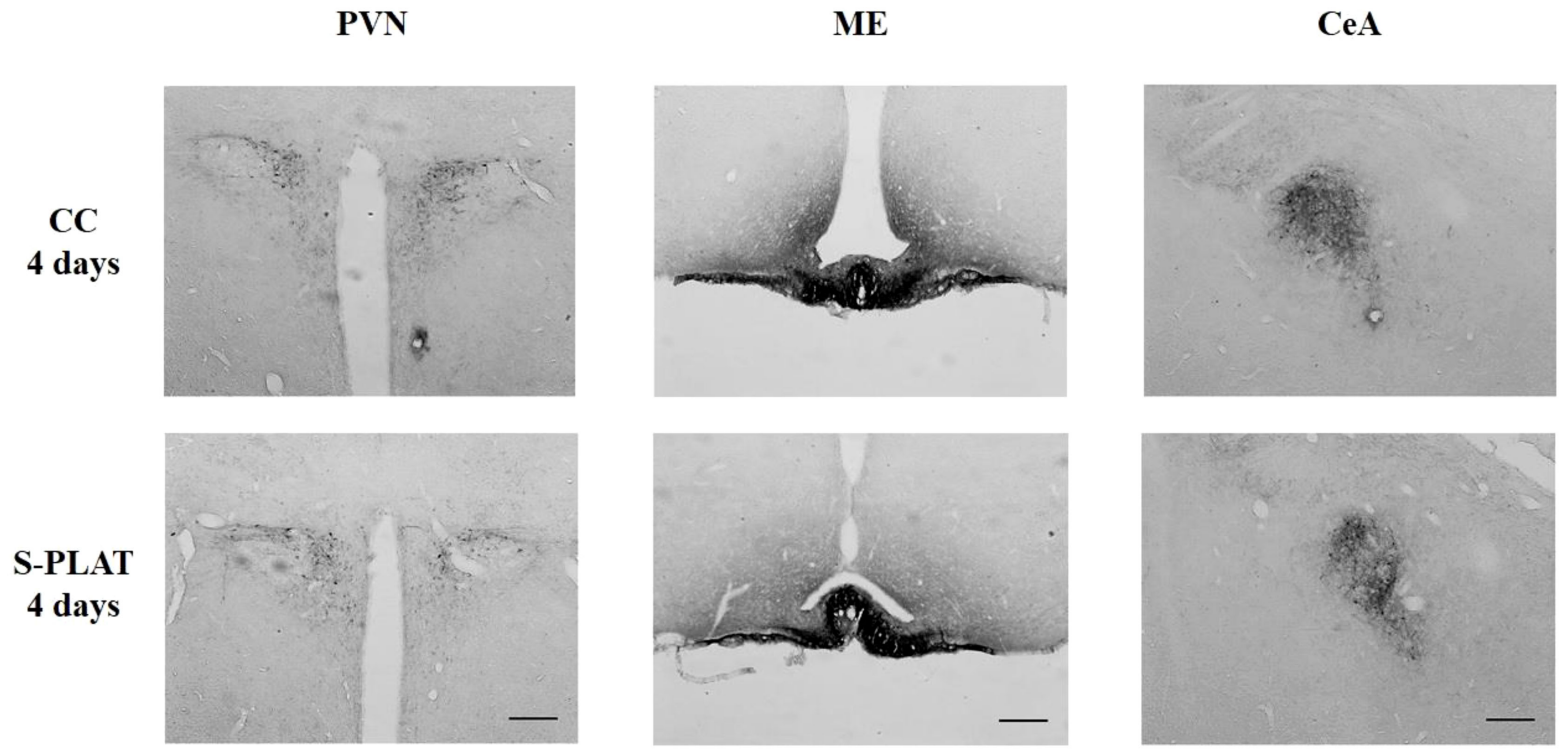
Representative photos of CRH immunocytochemistry in the PVN (left), in the external zone of the ME (middle) and CeA (right) of one control (CC) and one rat submitted to 4 days of paradoxical sleep deprivation (S-PLAT). Scale bar = 200 μm.

**Figure 5:**
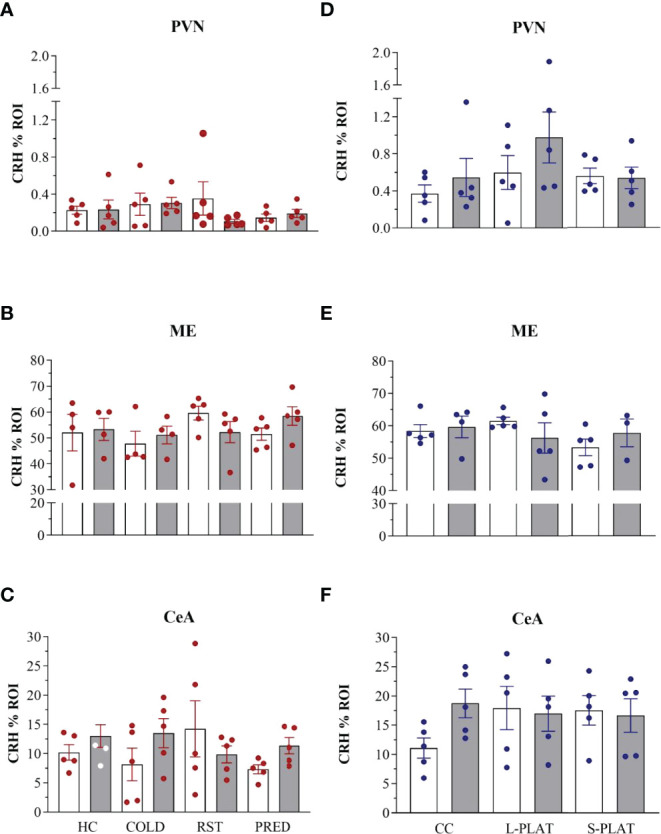
CRH-ir in the PVN, ME and CeA after several stressors **(A–C)** or after PSD **(D–F)**. Quantification of staining is shown for each individual and the columns represent the mean ± SEM of 4 to 5 rats/subgroup. HC, home cage; RST, restraint stress; PRED, predator odour; CC, container control; L-PLAT, large platform; S-PLAT, small platform. White columns represent single or 1-day exposure and grey columns, repeated or 4-day exposure.

ANOVA revealed no effects of stress on CRH staining in the PVN (F_3,32_ = 0.53, p = 0.66), ME (F_3,28_ = 0.97, p = 0.42) or CeA (F_3,32_ = 0.44, p = 0.72). No effects of frequency [PVN (F_1,32_ = 0.48, p = 0.49), ME (F_1,28_ = 0.14, p = 0.71), CeA (F_1,32_ = 1.28, p = 0.26)] or interaction between the factors [PVN (F_3,32_ = 1.11, p = 0.36), ME (F_3,28_ = 1.24, p = 0.31), CeA (F_3,32_ = 1.58, p = 0.21)] were observed. The data can be found in **Figures 4**, **5**.

In the sleep deprivation study, no effects on CRH-ir in the PVN (F_2,24_ = 1.92, p = 0.17), ME (F_2,21_ = 0.69, p = 0.51) or CeA (F_2,24_ = 0.49, p = 0.61) were detected. No effects of frequency [PVN (F_1,24_ = 1.56, p = 0.22), ME (F_1,21_ = 0.01, p = 0.95), CeA (F_1,24_ = 0.72, p = 0.40)] or interaction between the factors [PVN (F_2,24_ = 0.66, p = 0.53), ME (F_2,21_ = 1.25, p = 0.31), CeA (F_2,24_ = 1.57, p = 0.23)] were observed.

### Correlation Between CRH-ir in the PVN and CeA

Considering all subgroups, a positive correlation was observed between CRH in the PVN and in the CeA of animals in experiment 1 (r = 0.75; p < 0.001) and experiment 2 (r = 0.46; p < 0.05).

### ACTH

**Figure 6:**
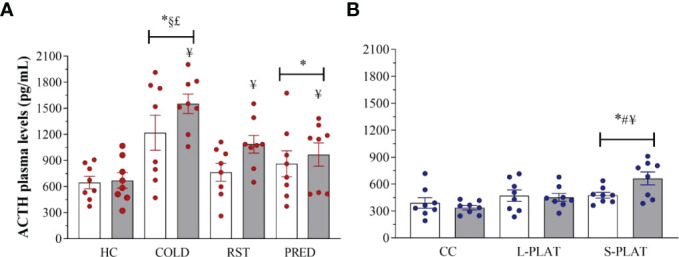
**(A)** Effect of different stressors and **(B)** of PSD on ACTH plasma levels. Data were plotted with individual values and the columns represent the mean ± SEM of 8 animals/subgroup. HC, home cage; RST, restraint stress; PRED, predator odour; CC, container control; L-PLAT, large platform; S-PLAT, small platform. White columns represent single or 1-day exposure and grey columns, repeated or 4-day exposure. * = different from respective control group; § = different from RST group; £ = different from PRED group; # = different from L-PLAT group; ¥ - different from single or from 1-day exposure.

Exposure to different stressors altered ACTH levels (**Figure 6A**). Main effects of Group (F_3,56 =_ 11.53; p < 0.001) and Frequency (F_1,62_ = 4.83; p < 0.05) were found. COLD led to the highest ACTH concentrations, compared to all other groups (p < 0.005). The ACTH response to PRED, but not to RST, was higher than that of HC animals (p < 0.05). Finally, ACTH concentrations were higher after repeated than single exposure (p < 0.05).

As for PSD, main effect of Group (F_2,42_ = 7.93; p < 0.05) and a nearly significant Group x Frequency interaction (F_2,42_ = 3.13; p = 0.054) were revealed (**Figure 6B**). The ACTH secretion after 4 days was higher in S-PLAT than in CC (p < 0.001) and L-PLAT groups (p < 0.04) as well as higher than after 1 day on small platforms (p < 0.02).

### Corticosterone

**Figure 7:**
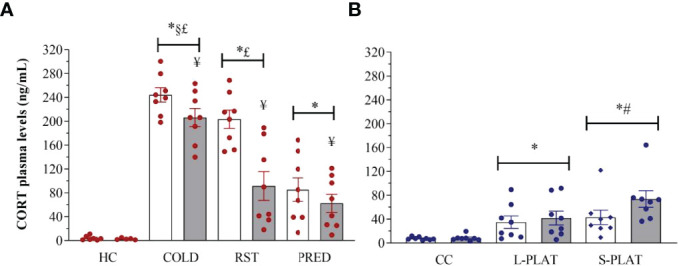
**(A)** Effect of different stressors and **(B)** of PSD on corticosterone plasma levels. Data were plotted with individual values and the columns represent the mean ± SEM of 13 animals/subgroup. HC, home cage; RST, restraint stress; PRED, predator odour; CC, container control; L-PLAT, large platform; S-PLAT, small platform. White columns represent single or 1-day exposure and grey columns, repeated or 4-day exposure. * = different from respective control group; § = different from RST group; £ = different from PRED group; # = different from L-PLAT group; ¥ - different from single or from 1-day exposure.

There were main effects of Group (F_3,52_ = 65.196, p < 0.0001) and Frequency (F_1,52_ = 14.196, p < 0.001), and a Group x Frequency interaction (F_3,52_ = 4.359, p < 0.01) after exposure of animals to different stressors (**Figure 7A**). The *post hoc* analysis showed that all stressors increased CORT levels above controls after 1 or 4 days of exposure, with COLD resulting in the highest levels (p’s < 0.001), followed by RST (p’s < 0.03) and PRED (p < 0.001). Moreover, only repeated RST led to lower CORT levels compared to a single exposure (p < 0.03).

In the sleep deprivation study (**Figure 7B**), ANOVA showed a main effect of Group (F_2,42_ = 13.115, p < 0.0001). Rats of the S-PLAT group exhibited higher CORT levels than L-PLAT (p < 0.05), which, in turn, had higher levels than CC group (p < 0.005).

### CORT : ACTH Ratio

**Figure 8:**
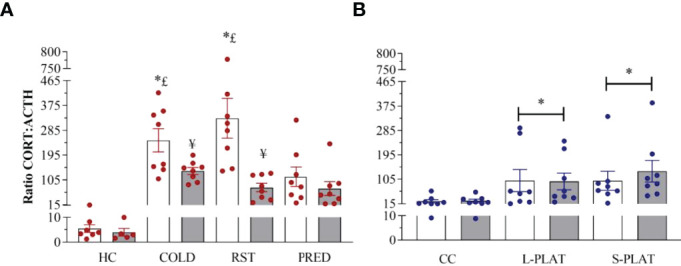
**(A)** Effect of different stressors and **(B)** of PSD on CORT : ACTH ratio. Data were plotted with individual values and the columns represent the mean ± SEM of 13 animals/subgroup. HC, home cage; RST, restraint stress; PRED, predator odour; CC, container control; L-PLAT, large platform; S-PLAT, small platform. White columns represent single or 1-day exposure and grey columns, repeated or 4-day exposure. * = different from respective control group; § = different from RST group; £ = different from PRED group; # = different from L-PLAT group; ¥ - different from single or from 1-day exposure.

Exposure to different stressors led to increased CORT : ACTH ratio (**Figure 8A**). Main effects of Group (F_3,52_ = 11.671, p < 0.0001), Frequency (F_1,52_ = 15.133, p < 0.0003) and Group x Frequency interaction (F_3,52_ = 4.398, p < 0.008) were shown. The *post hoc* test showed higher adrenal responsivity (p’s < 0.04) in singly exposed COLD and RST groups compared to both CTL and PRED groups. In addition, repeated exposure to COLD and RST reduced the CORT : ACTH ratio compared to the single exposure (p’s < 0.04).

CORT : ACTH ratio was increased above control values in rats of the S-PLAT and L-PLAT groups (p’s < 0.02), regardless of the length of exposure (main effect of Group: F_2,42_ = 5.18; p < 0.01) (**Figure 8B**).

## Discussion

This study showed that all stressors reduced body weight gain and increased plasma ACTH and CORT concentrations, albeit at different magnitudes. In addition, there was a reduction in the CORT response to repeated exposure to stressors, but not to prolonged PSD. Whilst no differences in CRH expression were found in the PVN, ME and CeA after exposure to any of the stressors, PSD was the only stimulus that reduced AVP expression in the PVN and increased it in the ME.

Several studies have shown an impairment in weight gain after repeated or chronic exposure to different stressors, such as cold (42), restraint (43–45), immobilization and foot shock (11, 46), PSD (47, 48), social defeat (49), chronic mild stress (50) and predator odour (51, 52). This outcome can be explained by the activation of the HPA axis (Harris 2014) and the catabolic role of CRH (53) and glucocorticoids (54). Among the classical stressors, cold was the one that led to greater impairment in this parameter, possibly as a direct effect of thermogenesis, with increased activation of the sympathetic nervous system and metabolic rate (55), oxygen consumption (56), activation of brown adipose tissue and increased ATP levels in different organs (57). PSD, in turn, caused an even greater loss in body weight, corroborating previous data from our and other groups (24, 47, 48, 58). This PSD effect can be explained by the intense catabolic state of the animals, evidenced by increased oxygen consumption and expression of uncoupling protein-1 in the brown adipose tissue (59), reduced body fat stores (33) and greater energy expenditure (33, 60). However, it is worth noticing that dwelling in the water container has an effect by itself. Nonetheless, methods of prolonged sleep deprivation or restriction, regardless of whether they involve water (17, 20) or not (61, 62), also result in body weight loss.

Reports indicate that under chronic stress situations, the HPA axis undergoes adaptation processes that include a shift from CRH- to AVP-driven ACTH release (63, 64). These adaptations were observed after 5 days-PSD for 3 h/day (65) or 14 days of 1 h/day restraint stress (66, 67) and long-term adjuvant-induced arthritis (68), i.e., longer protocols than the classical ones used in the present study. Among all the conditions used in this study, 4 days of PSD was the only one that decreased AVP-ir in the PVN and increased it in the ME, suggesting an augmented activity of this ACTH secretagogue. To the best of our knowledge, there is only one study that assessed the effects of 4 days of REMSD on AVP, showing a reduction of its mRNA in whole brain (69); however whole brain analysis has no parallel to assessments done in specific brain areas. There is a possibility that changes in AVP-ir could reflect an imbalance in the neuroendocrine control of the sleep-wake system, given that in the suprachiasmatic nucleus (SCN) this neuropeptide is involved in the circadian control of some functions, such as daily rhythm of the HPA axis (70) and spontaneous locomotor activity (71). Although we did not assess AVP-ir in the SCN, it is possible to speculate that the changes in sleep pattern imposed by sleep deprivation could be a major cause for activation of the AVP system. In addition, there is evidence of increased AVP expression in the PVN after acute exposure to cold and heat (72), higher AVP release within the PVN after social defeat (73) and increased AVP mRNA in the PVN after repeated exposure to cold and isolation stress (74). The AVP stress response seems to be neither specific nor limited to a stress category and response differences may be related to the stress protocol (intensity and duration), suggesting that the stressors used in the present study might not have been intense or prolonged enough to elicit a reliable AVP response. On the contrary, prolonged unremitting PSD, but not dwelling in the container or onto large platforms, reduced AVP expression in the PVN and increased it in the ME, indicating that long periods of sleep disruptions increased transport from the production (PVN) to the release (ME) sites and, therefore, resulted in greater activity of the AVPergic system, suggesting that AVP would likely be involved in PSD-induced higher ACTH levels after 4 days. The reason why PSD was the only stimulus to change AVP expression may be related to type of stress exposure; whilst the classical stressors lasted 30 or 60 min, PSD was continuously applied for 24 h or 96 h. Therefore, the animals were not allowed to recuperate from the stress. Evidence for this argument comes from a study showing that 6 h of PSD do not result in changes in the levels of AVP mRNA in the PVN (75). Furthermore, this effect indicates that sleep disruption is the main AVP-induced stimulus. Importantly, we analysed the magno- and parvocellular parts of the PVN and did not find group differences (data not shown). Thus, prolonged exposure to a stressor could recruit the AVP system to assist in the stress response, since none of the stimuli herein applied altered CRH expression in the PVN, ME and CeA. Previous studies using PSD show increased CRH expression in the PVN after 96 h (27) or 120 h of deprivation (28), indicating greater activity of this neuropeptide. Even after 72 h of PSD, lower CRH levels in the hypothalamus and higher CRH levels in the pituitary were reported (26). We note a discrepancy in CRH-ir between this and our previous findings (27), despite all experimental parameters being the same, except for blood sampling (daily tail nip in (27) and decapitation in the present study), the antibody vendor and method of quantification (counting of positive neurons in (27) and % ROI in the present study). In the CeA, CRH plays an important role in stress-adaptive responses (76, 77), mediating stress-induced anxiety-like behaviour through projections to the dorsolateral part of the bed nucleus of the stria terminalis (78). Several studies show higher CRH/CRH mRNA expression in the CeA after exposure to different stimuli (65, 79–81). Although we did not find any change in the CeA CRH expression, there was a positive correlation between CRH expression in the PVN and CeA in both experiments, indicating a possible participation of this structure in the stress response.

Cold, predator odour and PSD elicited a significant release of ACTH, replicating previous studies (82, 83). Predator odour-induced ACTH elevation is observed with several procedures, using natural and synthetic stimuli (84–88). Restraint did not produce a significant increase in ACTH values (p = 0.09), which agrees with studies using different protocols of this stressor (89, 90). Interestingly, the magnitude of the ACTH response to PSD was similar to that induced by predator odour, although a statistical difference from respective control groups was only obtained after 4 days of PSD. As for the CORT response, all classical stressors increased the release of the steroid, regardless of the stimulus and the frequency of application. A single exposure to cold or restraint led to the highest CORT levels, and this can be explained by the fact that physical and mixed stressors cause direct physiological changes for they require a rapid response of the stress systems (1, 14, 46). Even though a single session of PRED elicited a smaller CORT response, it was significantly above control values. The different CORT responses could be due to the nature of the stressor, but also to the duration of the session. Whilst COLD and RST lasted 1 h, PRED lasted 0.5 h and in all cases, animals were decapitated immediately after the stress session. Not surprisingly, repeated exposure to the classical stressors led to habituation of the CORT response, as shown in previous studies (91, 92). In contrast, 4 days of PSD led to greater CORT release, compared to the other conditions, replicating ours and others’ findings (18, 19, 21, 24, 27, 29, 31, 33, 35, 93–95). In addition, the distinct outcome observed between L-PLAT and S-PLAT groups indicates that loss of PS, rather than the environment to which animals are exposed, is the main factor leading to increased CORT release.

Compared to the single session, repeated exposure to the classical stressors increased ACTH levels, whilst the CORT response was slightly reduced, leading to a smaller CORT : ACTH ratio, although it was still higher than that in the control groups. This apparent dissociation between ACTH and CORT response to repeated stress indicates a reduction in adrenal sensitivity and may represent an adaptive response to prevent excessive CORT secretion. However, most studies report increased adrenal sensitivity to ACTH after chronic stressors of different natures, such as cold (96), restraint (97) and social instability (98). Importantly, the exposure to these stressors were much longer than in the present study and comparison of the CORT : ACTH ratio occurred at one time-point at the end of the stress protocol with non-stressed groups. Fourteen days of 2 h of restraint stress increases adrenal sensitivity to ACTH and decreases CORT clearance (97). Only a few studies report a longitudinal evaluation of ACTH and CORT levels, and CORT : ACTH ratio (99, 100) and our results are somewhat discordant. For instance, 1 h of immobilisation stress (a procedure that is more severe than restraint) results in similar levels of ACTH and CORT between 1 and 4 sessions, i.e., no change in adrenal sensitivity. Only by the 8th session, a reduction of ACTH and an increase of CORT levels are seen, compared to a single session, indicating increased adrenal sensitivity (99). Based on these findings, it is possible to suggest that short periods of repeated stress, as used in the present study, lead to an adaptive adjustment of the adrenal sensitivity to ACTH, which seems to be lost when the stressors endure for longer periods.

In contrast, 4 days of sleep deprivation led to increased ACTH and CORT levels compared to 1 day of exposure and to the control groups, resulting in similar ACTH : CORT ratio in L- and S-PLAT animals, both of which showed higher ratio than control values. The effect of sleep deprivation on ACTH : CORT ratio could be explained by a difference in adrenal ACTH receptors (MC2r), so that repeated stressors would produce downregulation, while prolonged exposure to PSD would cause upregulation of these receptors. In fact, a recent study showed that male mice have increased adrenal *MC2r* mRNA expression after 1 or 3 days of paradoxical sleep restriction (101).

Rodents are equipped to deal with several adverse situations that occur in nature. All of the stressors used in this study, are somehow related to natural conditions. Cold is a natural stressor but can be argued to be atypical because it is seasonal, and not intermittent as herein employed. Moreover, any rodent engages behavioural thermoregulation when temperature-challenged, as it evokes shivering and non-shivering thermogenesis (102). Restraint is one of the most employed stressors in the stress literature, but it may be artificial since rodents seldomly find themselves immobilised in nature unless caught in a narrow passageway. Predator odour, on the other hand, is a perfectly good natural stressor that does not have seasonal or diurnal differences. However, in nature, when rats detect predator scents, they resort to behaviours to avoid an encounter, such as fleeing and hiding. In our study, the rats did not have such an opportunity and their only option was to bury the petri dish with the shavings, which many of them did. Finally, PSD is not normally natural for rodents or other animals. They sleep when necessary and sleep is usually not restricted in any way, except when they need to flee and hide from a predator or in case of natural disasters. In any way, these situations represent challenges to the animals and, therefore, recruit the HPA axis and the autonomic nervous system to meet the demands imposed.

**Figure 9:**
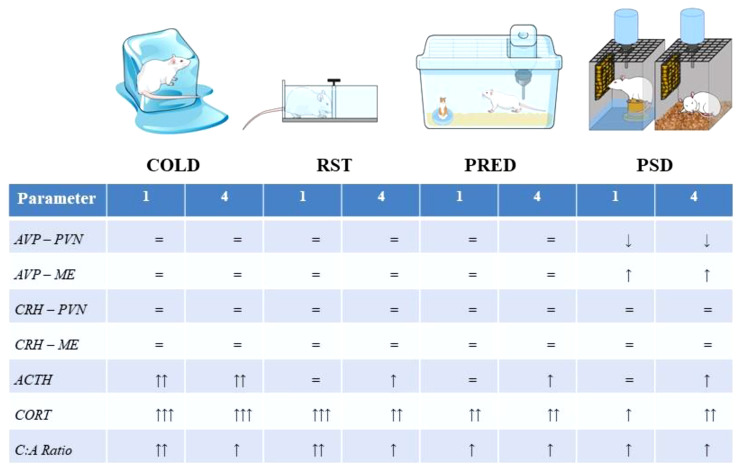
Representation of the stressors used in the present study and the magnitude of the response at each level of the HPA axis. The Abbreviations: ACTH, adrenocorticotropic hormone; AVP, arginine-vasopressin; C:A ratio, CORT, ACTH ratio; COLD, cold stress; CORT, corticosterone; CRH, corticotropin releasing hormone; ME, median eminence; PRED, predator scent; PVN, paraventricular nucleus of the hypothalamus, RST, restraint stress.

A comparative summary of the HPA axis response to the stimuli is presented in **Figure 9**. A qualitative comparison reveals that the pattern and magnitude of PSD stress response resembles more those induced by exposure to predator odour than by cold and restraint stressors, even though PSD methodology involves the association of typically physical stressors (contact with water and restricted movement). This is not surprising in view of the negative impact of sleep restriction on mental health in humans (103) and on emotional behaviours in rats (104). Finally, PSD was the only stimulus which altered AVP expression in the hypothalamus, an effect that could explain the sustained high ACTH secretion after 4 days of sleep deprivation.

## Data Availability Statement

The raw data supporting the conclusions of this article will be made available by the authors, without undue reservation.

## Ethics Statement

The animal study was reviewed and approved by Committee on the Use of Experimental Animals - Universidade Federal de São Paulo.

## Author Contributions

DM, RM and DS designed the study. DM collected the data. DM and GH run the immunohistochemistry study. DM and DS analyzed the data and wrote the manuscript. GH and MK edited and helped writing the manuscript. All authors contributed to the article and approved the submitted version.

## Funding

DM received a Ph.D. fellowship and an internship at Dr. Gloria Hoffman’s laboratory from Fundação de Amparo à Pesquisa do Estado de São Paulo (FAPESP). DS is the recipient of a Research Fellowship from Conselho Nacional de Desenvolvimento Científico e Tecnológico (grant # 302608/2019-2). Payment for this publication was provided by FAPESP (grant # 2019/21980-0).

## Conflict of Interest

The authors declare that the research was conducted in the absence of any commercial or financial relationships that could be construed as a potential conflict of interest.

## Publisher’s Note

All claims expressed in this article are solely those of the authors and do not necessarily represent those of their affiliated organizations, or those of the publisher, the editors and the reviewers. Any product that may be evaluated in this article, or claim that may be made by its manufacturer, is not guaranteed or endorsed by the publisher.
