# Supplementary figures and images for: The Pituitary-Adrenal Response to Paradoxical Sleep Deprivation Is Similar to a Psychological Stressor, Whereas the Hypothalamic Response Is Unique
Source: Front Endocrinol (Lausanne). 2022 Jul 8;13:885909. doi: 10.3389/fendo.2022.885909 (PMC9308007; doi:10.3389/fendo.2022.885909)

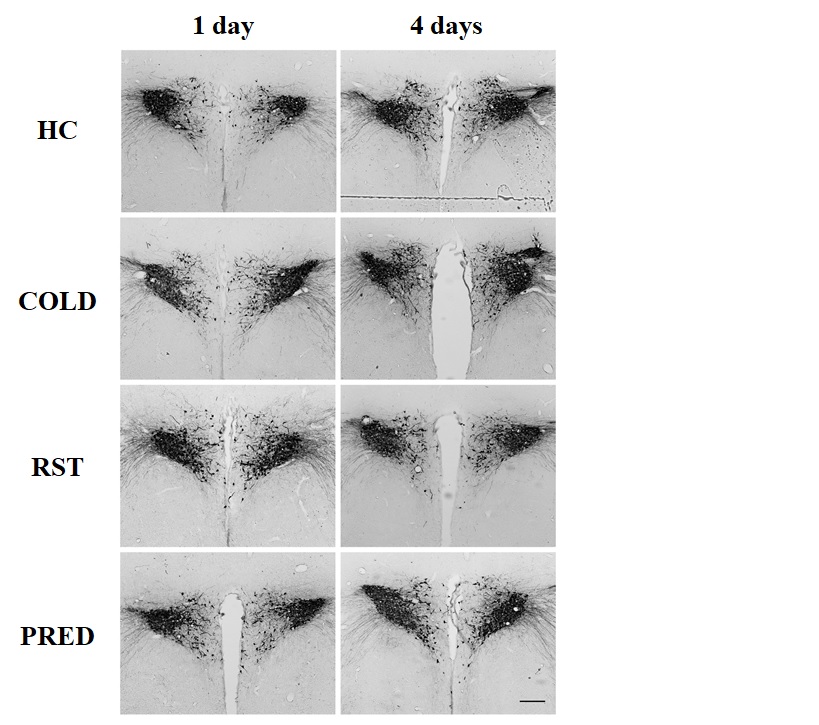

Supplement: Supplementary file 1 [file Image_1.jpeg]

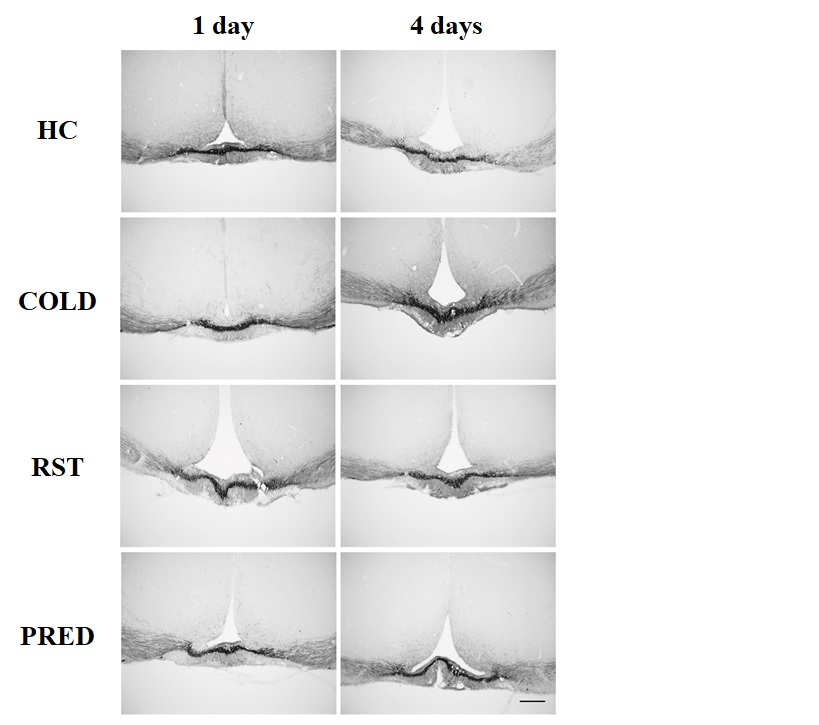

Supplement: Supplementary file 2 [file Image_2.jpeg]

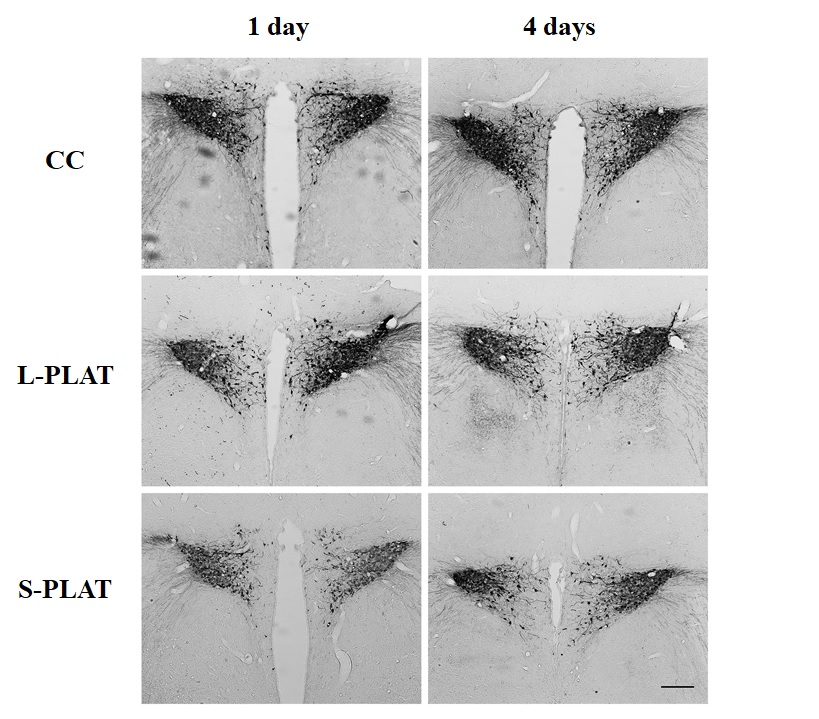

Supplement: Supplementary file 3 [file Image_3.jpeg]

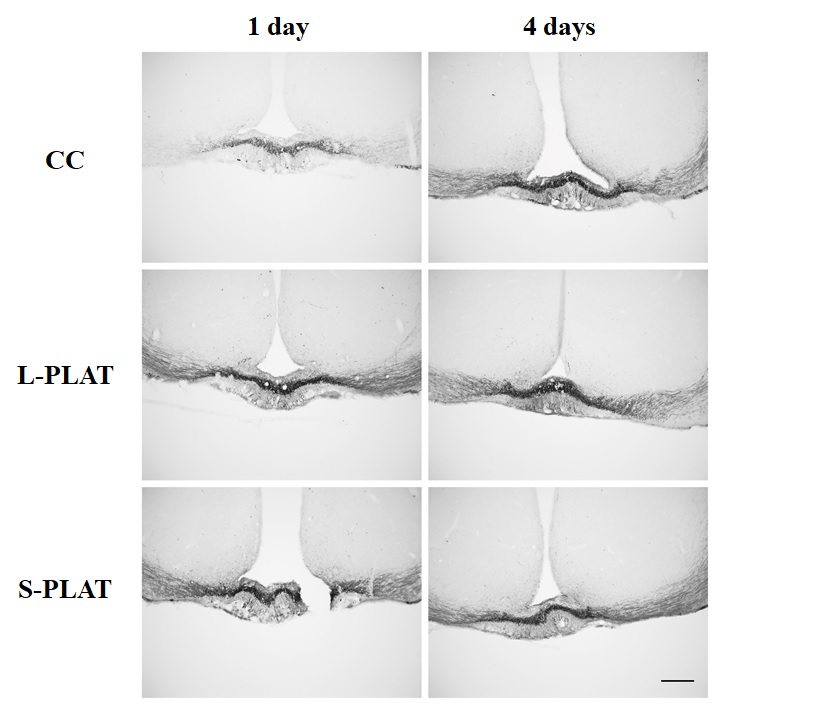

Supplement: Supplementary file 4 [file Image_4.jpeg]

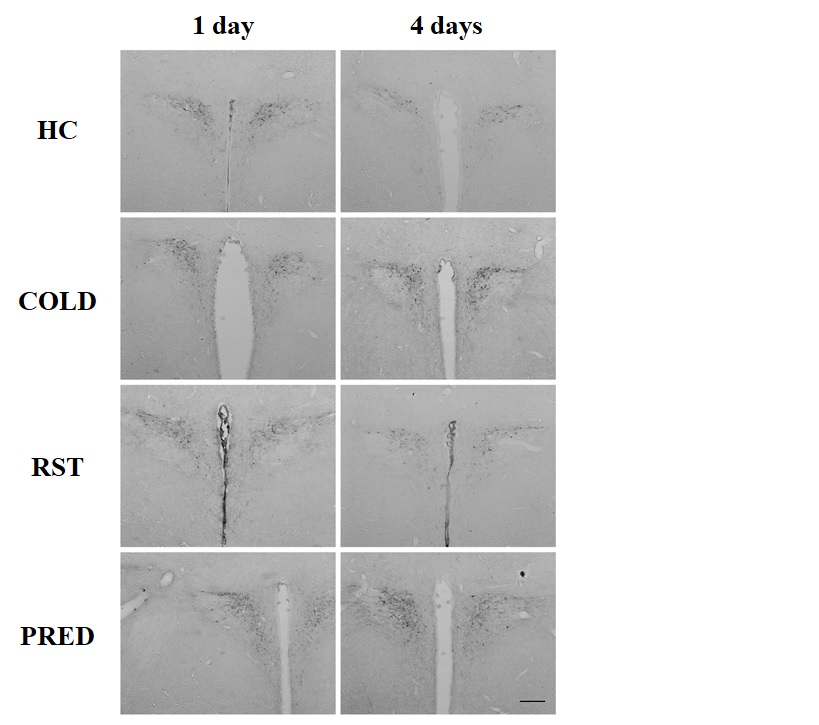

Supplement: Supplementary file 5 [file Image_5.jpeg]

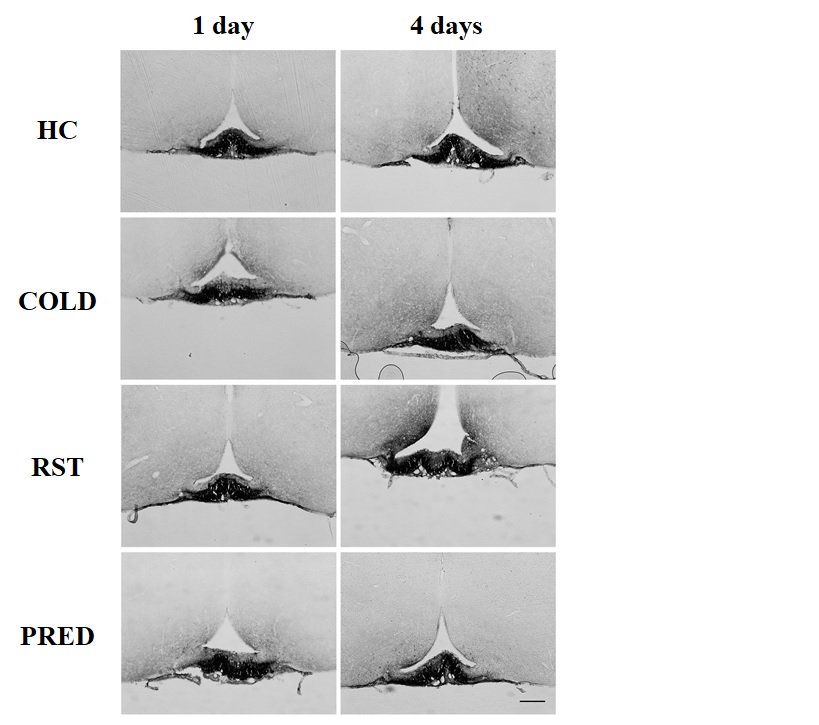

Supplement: Supplementary file 6 [file Image_6.jpeg]

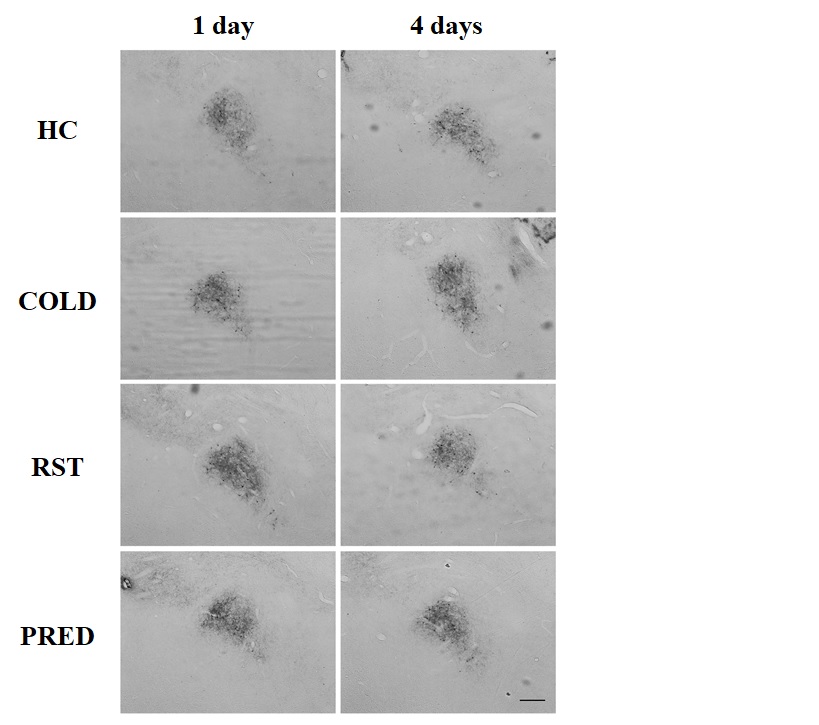

Supplement: Supplementary file 7 [file Image_7.jpeg]

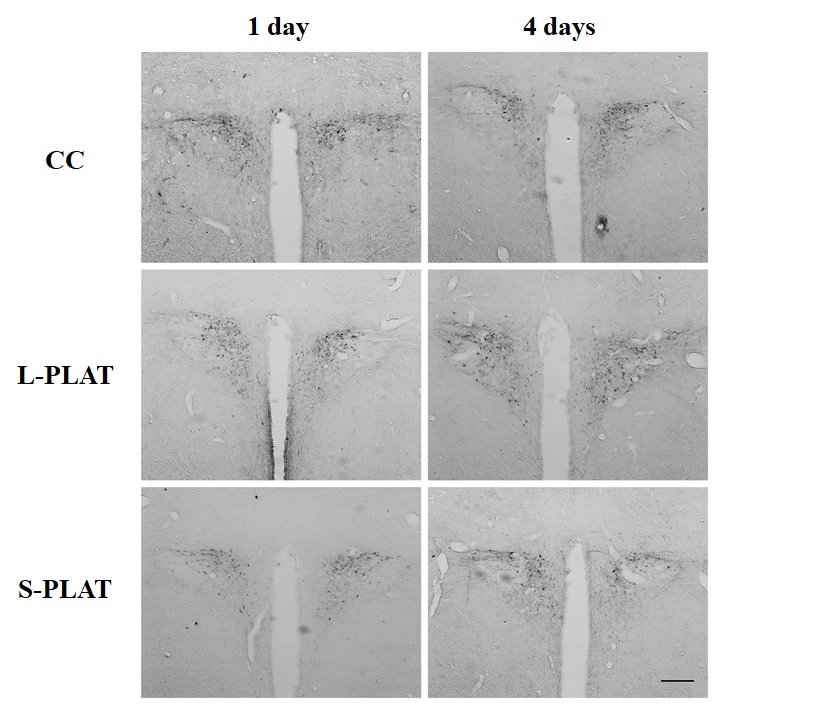

Supplement: Supplementary file 8 [file Image_8.jpeg]

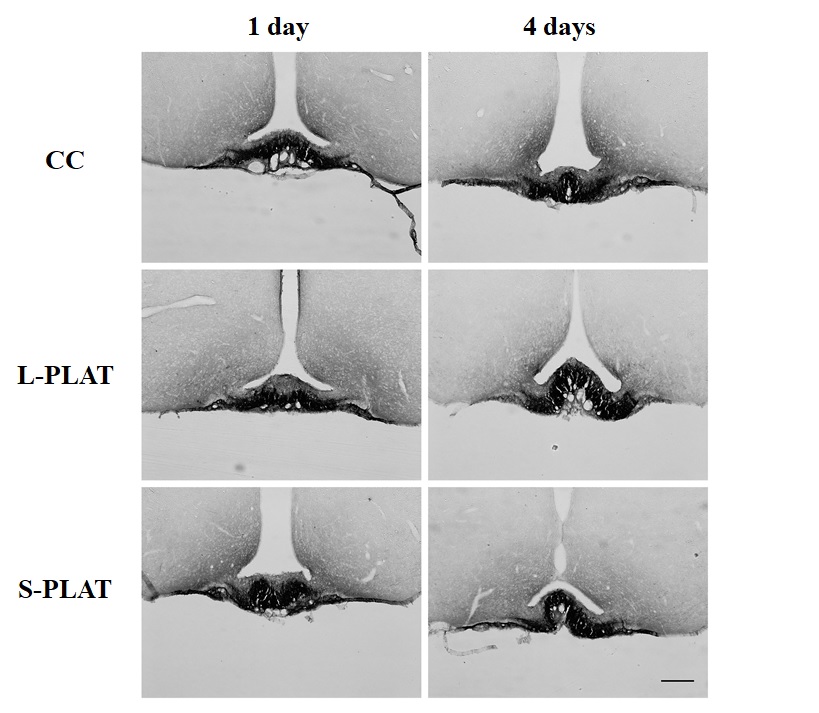

Supplement: Supplementary file 9 [file Image_9.jpeg]

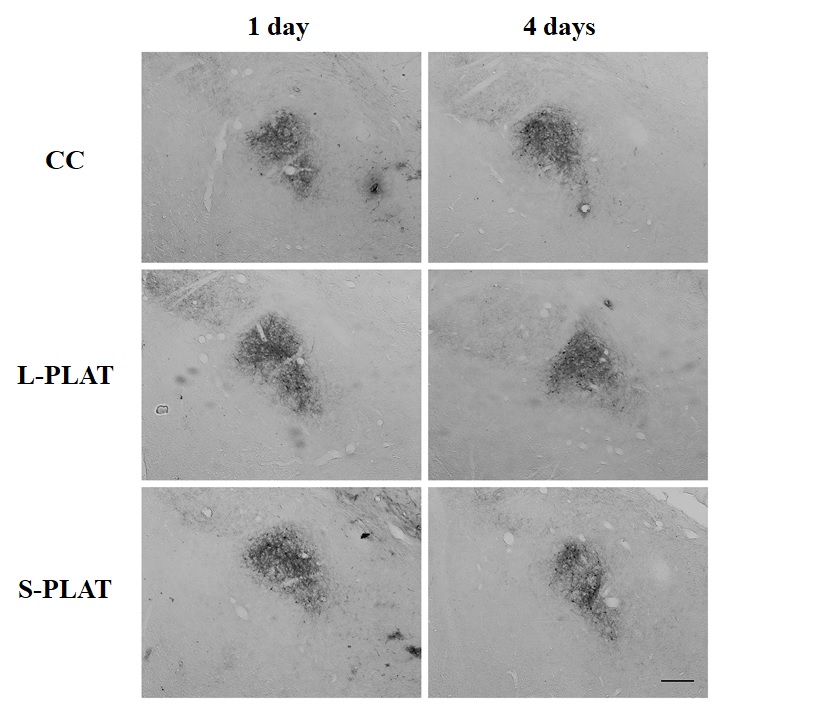

Supplement: Supplementary file 10 [file Image_10.jpeg]
